# The Impact of Metabolic and Bariatric Surgery on Nutritional Status and Sarcopenia: A Narrative Overview of Underlying Mechanisms, Clinical Implications, and Prevention Strategies

**DOI:** 10.3390/nu18172923

**Published:** 2026-09-07

**Authors:** Ligia J. Dominguez, Francesco Saverio Ragusa, Nicola Veronese, Giovanna Di Bella, Stefano Ciriminna, Flavia Seminara, Salvatore Maria Baio, Mario Barbagallo

**Affiliations:** 1 Department of Psychology, Educational Science and Human Movement, University of Palermo, 90133 Palermo, Italy; 2 Pro.M.I.S.E. Department “Giuseppe D’Alessandro”, University of Palermo, 90127 Palermo, Italy; mario.barbagallo@unipa.it; 3 Geriatric Unit, Department of Internal Medicine and Geriatrics, “Paolo Giaccone” University Hospital, 90100 Palermo, Italy; francescosaverioragusa@gmail.com (F.S.R.); giovannadibella79@libero.it (G.D.B.); steciri@gmail.com (S.C.); semifla94@gmail.com (F.S.); salvatoremariabaio@gmail.com (S.M.B.); 4 Faculty of Medicine, Saint Camillus International University of Health Sciences, 00131 Rome, Italy; nicola.veronese@unicamillus.org

**Keywords:** bariatric surgery, metabolic surgery, sarcopenia, sarcopenic obesity, body composition, nutritional deficiencies, protein intake, muscle mass, muscle performance

## Abstract

Bariatric surgery is the most effective treatment for severe obesity, leading to significant and sustained weight loss and improvement in metabolic comorbidities. However, it is also associated with substantial changes in body composition and nutritional status, including the risk of sarcopenia and sarcopenic obesity. This review aims to summarize current evidence on the interplay between metabolic and bariatric surgery, nutritional alterations, and skeletal muscle health. The recent literature highlights a high and increasing prevalence of muscle loss after surgery, driven by reduced protein intake, micronutrient deficiencies, hormonal changes, and decreased mechanical loading. Despite these challenges, improvements in metabolic health and physical function are often observed, suggesting a complex relationship between muscle mass and functional outcomes. A multidisciplinary approach, including nutritional optimization and structured exercise interventions, is essential to mitigate muscle loss and function and improve long-term outcomes. Further research is needed to standardize diagnostic criteria and develop targeted strategies for prevention and management.

## 1. Introduction

Obesity represents a major global health concern, with its prevalence continuing to rise worldwide and posing a substantial burden on healthcare systems. It is strongly associated with an increased risk of cardiometabolic diseases, including type 2 diabetes, cardiovascular disease, and certain cancers, as well as functional impairment and reduced quality of life [1,2]. In this context, metabolic and bariatric surgery (MBS) has emerged as the most effective long-term treatment for severe obesity, producing significant and sustained weight loss and leading to improvement or remission of obesity-related comorbidities [3,4,5,6]. Current international guidelines recommend MBS not only for weight reduction but also as a metabolic intervention for patients with obesity and associated diseases [5].

Despite these well-established benefits, MBS induces profound changes in nutritional status and body composition. The rapid weight loss observed after surgery is characterized not only by a substantial reduction in fat mass but also by a concomitant and clinically relevant loss of lean body mass [7]. It has been estimated that up to 20–30% of total weight loss may derive from fat-free mass, depending on the surgical procedure and postoperative management [6,8,9]. This loss of skeletal muscle is of particular concern, as it may negatively affect metabolic health, physical function, and long-term weight maintenance [10,11].

In this regard, increasing attention has been directed toward the development of sarcopenia following MBS. Sarcopenia is a progressive and generalized skeletal muscle disorder characterized by the loss of muscle mass, strength, and physical performance [12]. While traditionally associated with aging, it is now recognized in a variety of clinical conditions, including obesity and weight loss interventions [11,13]. Emerging evidence suggests that sarcopenia is relatively common after MBS, with prevalence varying widely depending on diagnostic criteria and timing of assessment [8,14,15]. Notably, muscle loss appears to be more pronounced during the first postoperative year but may persist or even worsen over time, particularly in the absence of adequate nutritional and physical activity interventions [14,15].

In parallel, the concept of sarcopenic obesity—defined by the coexistence of excess adiposity and reduced muscle mass and function—has gained increasing recognition as a clinically relevant phenotype [13,16]. This condition is associated with worse metabolic outcomes, increased disability, and higher mortality compared to either obesity or sarcopenia alone [16,17]. Patients undergoing MBS may be particularly vulnerable to this condition, especially when significant muscle loss accompanies incomplete fat mass reduction or weight regain [8].

The pathophysiology of sarcopenia after MBS is complex and multifactorial. Surgical procedures, particularly those with a malabsorptive component, can lead to reduced dietary intake, altered gastrointestinal anatomy, and impaired digestion and absorption of nutrients [11,18,19]. Consequently, patients are at high risk of developing deficiencies in protein, vitamins (such as vitamin D, A, and B12), and minerals (including iron, magnesium, and calcium), all of which play essential roles in muscle metabolism and function [5,19,20,21,22]. Inadequate protein intake, in particular, contributes to a negative nitrogen balance and promotes muscle protein breakdown while impairing muscle protein synthesis [22,23,24]. Additionally, rapid weight loss induces a catabolic state characterized by hormonal and metabolic adaptations that further exacerbate muscle loss. Changes in gut-derived hormones, insulin sensitivity, and inflammatory mediators have been implicated in the regulation of muscle metabolism following bariatric surgery [11,25,26]. Moreover, the reduction in mechanical loading due to substantial weight loss may decrease the anabolic stimulus required to maintain muscle mass and strength [27]. Physical inactivity, which is common in this population, further compounds these effects [28].

Given these considerations, understanding the interplay between MBS, nutritional status, and sarcopenia is essential for optimizing patient outcomes. Early identification of individuals at risk, along with targeted interventions such as adequate protein intake, micronutrient supplementation, and structured exercise programs, may help mitigate muscle loss and preserve functional capacity [29,30]. Therefore, this narrative review aims to provide an updated overview of the current evidence on the effects of MBS on nutrition and sarcopenia, with particular emphasis on underlying mechanisms, clinical implications, and potential strategies for prevention and treatment.

## 2. Search Methods

For the present narrative review, PubMed/MEDLINE was searched for English-language, peer-reviewed articles published from 1 January 2020 through 1 July 2026. The search combined terms related to metabolic and bariatric surgery with terms related to nutritional status and skeletal muscle health, using the following strategy: (‘bariatric surgery’ OR ‘metabolic surgery’) AND (‘nutrition’ OR ‘nutritional deficiency’ OR ‘sarcopenia’ OR ‘sarcopenic obesity’ OR ‘body composition’ OR ‘protein intake’ OR ‘muscle mass’ OR ‘muscle performance’). Longitudinal studies, clinical studies, narrative reviews, systematic reviews, meta-analyses, umbrella reviews, consensus statements, and clinical guidelines were considered. Reference lists of relevant publications were hand-searched, and forward citation searches were used to identify additional pertinent studies. Earlier landmark publications were retained when required for historical definitions, diagnostic criteria, or foundational evidence. Case reports, case series, editorials, letters to the editor, comments, short reports, short communications, and perspectives were excluded.

## 3. Types of Metabolic and Bariatric Surgery

The terminology and classification of bariatric surgery have evolved substantially in parallel with the increasing recognition that these procedures exert effects extending well beyond simple gastric restriction or intestinal malabsorption. Historically, bariatric procedures were broadly categorized as restrictive, malabsorptive, or combined restrictive–malabsorptive operations. Although this terminology remains useful for describing certain anatomical characteristics, it is increasingly inadequate for contemporary MBS, because many modern procedures combine gastric resection, alterations in gastrointestinal transit, changes in nutrient sensing, enteroendocrine signaling, bile-acid metabolism, and intestinal adaptation. Recent classifications therefore increasingly emphasize anatomical configuration and physiological/metabolic mechanisms rather than restriction and malabsorption alone [31,32].

A recently proposed anatomical classification divides bariatric procedures into resectional bariatric surgery (RBS) and non-resectional bariatric surgery (NRBS). Resectional procedures include operations in which a substantial portion of the stomach is removed, whereas non-resectional procedures preserve the stomach. These two major groups can subsequently be subdivided according to whether the operation involves the stomach alone, the stomach and small intestine, or the small intestine alone. Within this framework, sleeve gastrectomy (SG) represents a resectional, isolated gastric procedure; Roux-en-Y gastric bypass (RYGB) and one-anastomosis gastric bypass (OAGB) represent non-resectional gastrointestinal bypass procedures; while procedures such as single-anastomosis duodeno-ileal bypass with sleeve gastrectomy (SADI-S) and single-anastomosis sleeve ileal bypass (SASI) combine gastric resection with intestinal bypass. This classification has the advantage of describing the actual anatomical modifications produced by contemporary operations and may be particularly useful for surgical documentation, communication, and the management of conditions involving the remnant stomach or altered gastrointestinal anatomy [32] (Table 1).

A complementary metabolic/physiological classification proposed in 2025 recognizes that the effects of MBS extend beyond restriction and malabsorption. It categorizes procedures according to their effects on gastric anatomy, intestinal transit, biliopancreatic diversion, and enteroendocrine and bile-acid signaling, incorporating concepts such as gastric resection, gastrointestinal bypass, transit bipartition, duodenal bipartition, and common-channel length. The framework also includes the concepts of a metabolically functional stomach, wide anastomosis, and isolated or hybrid duodenal bipartition [33].

Under this contemporary concept, SG should not be regarded simply as a restrictive operation. In addition to reducing gastric volume, SG modifies gastric emptying and nutrient delivery and is associated with important endocrine and metabolic changes. RYGB combines gastric restriction with gastrointestinal bypass and substantial modification of nutrient flow. OAGB similarly combines gastric pouch creation with a single gastrojejunal anastomosis and a variable degree of intestinal bypass. Its increasing recognition as an established metabolic procedure is reflected by the 2024 American Society for Metabolic and Bariatric Surgery (ASMBS) position statement, which reviewed its role in obesity and metabolic disease [31].

The distinction between OAGB and RYGB is particularly relevant to contemporary classification. OAGB is increasingly considered a major metabolic procedure rather than merely a technical variation in gastric bypass [31]. A 5-year randomized extension of the YOMEGA trial demonstrated sustained efficacy of OAGB compared with RYGB, although the two procedures have different nutritional and gastrointestinal considerations [34]. More recent systematic reviews and meta-analyses have also demonstrated substantial efficacy of OAGB for weight loss and type 2 diabetes, supporting its inclusion among the principal contemporary metabolic procedures [35].

Similarly, SADI-S occupies an important position in the modern classification because it combines SG with a single duodeno-ileal anastomosis and a relatively long bypassed intestinal segment. It therefore cannot be adequately described simply as either “restrictive” or “malabsorptive.” Its metabolic effects reflect the interaction between gastric resection, preservation of pyloric and duodenal passage, altered nutrient exposure, and intestinal bypass. The emergence of SADI-S and other single-anastomosis procedures illustrates why a mechanism-based classification is increasingly preferable to the traditional three-category model [32].

Another important development is the increasing recognition of transit-bipartition procedures, including SG with transit bipartition (SGTB). These procedures are designed to modify intestinal nutrient transit while maintaining a substantial proportion of physiological gastrointestinal continuity. Emerging international and national consensus documents have identified OAGB, SADI-S and SGBT as important emerging or established metabolic operations, although the level of evidence and long-term follow-up differs between procedures [32,33].

The recently proposed metabolic/physiological classification should therefore be considered complementary to, rather than a replacement for, existing classification systems [32]. While an anatomical classification defines the structural modifications introduced by surgery, a physiological and metabolic classification seeks to characterize how these alterations affect gastrointestinal function and metabolic pathways, thereby accounting for the diverse metabolic and nutritional consequences associated with each surgical procedure. However, the 2025 new classification represents a proposed conceptual framework rather than a universally adopted system or an official IFSO/ASMBS classification, and it should not be considered a replacement for the traditional nomenclature. The IFSO itself has published ethical standards that must be followed [36], especially for new procedures where there is still insufficient evidence of their risks, benefits, and overall safety, and therefore they cannot yet be considered established surgical practice. The discussion below emphasizes SG, RYGB, OAGB, and SADI-S because of their contemporary clinical relevance. Older or less frequently performed operations are discussed more briefly where they provide important historical context or illustrate procedure-specific nutritional risks.

### 3.1. Sleeve Gastrectomy and Gastric-Only Procedures

Sleeve gastrectomy is currently the most frequently performed metabolic and bariatric procedure worldwide [37]. Although historically categorized as a restrictive operation, this terminology does not adequately reflect its mechanisms. In addition to reducing gastric volume and promoting early satiety, SG alters gastric emptying, nutrient delivery, ghrelin secretion, enteroendocrine signaling, and other metabolic pathways [38]. The operation involves resection of a large portion of the stomach, leaving a narrow gastric sleeve. Nutritional complications may arise through reduced dietary intake, intolerance to nutrient-dense foods, persistent or recurrent vomiting, altered gastric acid secretion, and accelerated gastric emptying [38,39]. Thus, SG should be described according to its anatomical and physiological effects rather than as a purely or primarily restrictive procedure.

Adjustable gastric banding (AGB), now performed infrequently, is an example of a gastric-only procedure in which weight loss is driven predominantly by limitation of oral intake. Nutritional complications may nevertheless occur and are commonly related to reduced food variety, maladaptive eating behaviors, persistent vomiting, food intolerance, or poor adherence to supplementation protocols [40].

Overall, gastric-only procedures can produce substantial weight loss and metabolic benefits, but they remain associated with clinically relevant nutritional risks. Adequate dietary education, routine laboratory screening, prompt management of vomiting or food intolerance, and multidisciplinary postoperative care are therefore essential components of long-term management [38].

### 3.2. Gastrointestinal Bypass and Procedures with Greater Malabsorptive Potential

Gastrointestinal bypass procedures and operations with a greater malabsorptive component achieve weight loss and metabolic improvement through combinations of altered gastrointestinal anatomy, reduced nutrient contact with proximal intestine, modified nutrient flow, and endocrine/metabolic effects. The extent of nutrient malabsorption varies considerably by procedure and by limb or common-channel length, and contemporary operations should therefore not be reduced to a single ‘malabsorptive’ category [11].

Among currently relevant procedures, RYGB and OAGB combine gastric pouch creation with intestinal bypass, whereas SADI-S combines sleeve gastrectomy with duodeno-ileal bypass. Conventional BPD and BPD with duodenal switch (BPD-DS) are historically important procedures with substantial malabsorptive potential but are now performed infrequently in routine bariatric practice. SG, in contrast, generally produces minimal direct nutrient malabsorption; its nutritional effects arise mainly from reduced intake, altered gastric physiology, intolerance, vomiting, and metabolic adaptations [41,42].

The degree of nutrient malabsorption depends on several anatomical and physiological factors, including exclusion of the duodenum and proximal jejunum, delayed mixing of food with bile acids and pancreatic enzymes, shortening of the common channel, and changes in gastrointestinal hormones and microbiota [43]. These effects are most pronounced in procedures with longer bypassed segments or shorter common channels. RYGB generally produces moderate nutrient malabsorption, whereas more hypoabsorptive configurations such as BPD-DS and some SADI-S or OAGB variants may carry substantially greater nutritional risk [42].

## 4. Nutritional Deficiencies After Bariatric Surgery

Nutritional deficiencies are a well-recognized consequence of MBS due to reduced intake, altered digestion, and, in some procedures, intestinal malabsorption. Macronutrient deficiencies, particularly inadequate protein intake, can contribute to loss of lean body mass, impaired wound healing, and reduced immune function. Micronutrient deficiencies are also common and may involve iron, vitamin B12, folate, calcium, and fat-soluble vitamins (A, D, E, and K), depending on the surgical technique and adherence to supplementation. These deficiencies can lead to anemia, bone demineralization, neurological symptoms, and metabolic complications if not properly monitored and treated. Therefore, lifelong nutritional surveillance and individualized supplementation are essential to prevent clinically significant deficiencies and optimize long-term outcomes after MBS. Table 2 summarizes the most common nutritional and metabolic consequences of the different procedures, including expected weight loss, lean-mass reduction, protein-deficiency risk, micronutrient deficiencies, recommended laboratory monitoring, and long-term nutritional management. These values are approximate because substantial variability has been reported across studies.

### 4.1. Macronutrient Malabsorption

Protein-energy malnutrition is less common after gastric-only procedures such as SG than after procedures with substantial intestinal bypass, but inadequate protein intake may still occur, especially during the early postoperative period when oral intake is markedly reduced. Inadequate protein consumption can contribute to sarcopenia, hair loss, impaired wound healing, and reduced functional capacity [49]. Therefore, international guidelines recommend prioritizing high-biological-value protein sources and maintaining lifelong nutritional surveillance following MBS [5,20]. Inadequate dietary intake, vomiting, and poor adherence to nutritional recommendations further exacerbate this condition [11].

Fat malabsorption is a characteristic consequence of procedures with a substantial malabsorptive component. Reduced lipid absorption contributes to weight loss but also predisposes patients to steatorrhea, diarrhea, bloating, and deficiencies of fat-soluble vitamins (A, D, E, and K). Persistent steatorrhea may significantly impair quality of life and lead to social limitations [50].

Carbohydrate malabsorption is generally less pronounced but may contribute to altered glycemic regulation and changes in colonic fermentation. Increased delivery of undigested nutrients to the colon can alter microbiota composition and increase bacterial fermentation activity [43,51].

### 4.2. Micronutrient Deficiencies

Micronutrient deficiencies are among the most clinically relevant long-term complications of MBS (Table 2). The prevalence and severity depend on the type of procedure, preoperative nutritional status, adherence to supplementation, and duration of follow-up.

• Vitamin B12 and Folate Deficiency: Results from reduced intrinsic factor production, decreased gastric acid secretion, and bypass of the terminal digestive pathways necessary for cobalamin release from food proteins. Neurological manifestations may include peripheral neuropathy, cognitive impairment, and megaloblastic anemia. Folate deficiency may coexist and contribute to hematologic abnormalities [20,38,52].
• Vitamin B1 (Thiamine) Deficiency: Thiamine deficiency is among the most clinically important water-soluble vitamin deficiencies after MBS and may occur after SG as well as after RYGB or other bypass procedures, particularly in the setting of persistent or recurrent vomiting, markedly reduced oral intake, or rapid weight loss. If untreated, it may cause wet, dry, or cerebral beriberi, with cardiovascular or neurological manifestations ranging from confusion to psychosis. Deficiency may develop within the first months after surgery and can progress rapidly. Because neurological injury may become irreversible, thiamine treatment should not be delayed when deficiency is strongly suspected, especially in patients with persistent vomiting or neurological manifestations [39].
• Iron Deficiency: Ferropenic anemia is one of the most common postoperative complications, particularly after RYGB and BPD-DS. Iron absorption normally occurs in the duodenum and proximal jejunum, which are excluded in these procedures. Reduced gastric acid secretion further impairs iron bioavailability. Menstruating women are especially vulnerable [52].
• Calcium and Vitamin D Deficiency: Calcium is predominantly absorbed in the duodenum, making impaired calcium uptake a common consequence of malabsorptive bariatric procedures. Inadequate vitamin D status further reduces calcium absorption and may promote secondary hyperparathyroidism, osteopenia, and osteoporosis
• Calcium and Vitamin D Deficiency: Calcium is predominantly absorbed in the duodenum, making impaired calcium uptake a common consequence of procedures with a substantial malabsorptive component. Inadequate vitamin D status further reduces calcium absorption and may promote secondary hyperparathyroidism, osteopenia, and osteoporosis [53]. Long-term studies, particularly in patients undergoing BPD-DS, have reported persistent vitamin D insufficiency and elevated parathyroid hormone concentrations despite supplementation [41]. Notably, vitamin D deficiency is also frequent before surgery, highlighting the importance of assessing and correcting pre-existing deficiencies to minimize postoperative bone loss and metabolic complications [54].
• Fat-Soluble Vitamin Deficiencies: Deficiencies of vitamins A, E, and K are especially associated with BPD-DS due to severe fat malabsorption. Vitamin A deficiency may lead to night blindness and xerophthalmia, while vitamin K deficiency can impair coagulation. Meta-analytic data indicate that more than one-quarter of patients undergoing BPD-DS develop vitamin A deficiency during long-term follow-up [41].
• Trace Elements and Magnesium: Deficiencies of zinc, selenium, copper, and magnesium have also been described after procedures with a substantial malabsorptive component [20,39]. Copper deficiency may cause anemia and neurological syndromes that mimic vitamin B12 deficiency. Zinc deficiency can impair immune function and wound healing, whereas selenium deficiency may contribute to cardiomyopathy and myopathy [55].

### 4.3. Gastrointestinal and Metabolic Adaptation

Emerging evidence suggests that the gastrointestinal tract undergoes substantial adaptation after MBS. Hormonal changes involving glucagon-like peptide-1 (GLP-1), peptide YY, bile acids, and gut microbiota contribute not only to weight loss but also to modifications in nutrient handling and metabolism. These adaptive mechanisms may partially compensate for malabsorption but can also influence long-term nutritional status [39,43]. Changes in gut microbiota after surgery with a substantial malabsorptive component are increasingly recognized as contributors to both metabolic benefits and nutritional complications. Altered microbial fermentation and nutrient metabolism may influence vitamin synthesis, intestinal permeability, and inflammatory responses [56].

### 4.4. Nutritional Monitoring and Supplementation

Because nutritional complications may develop years after surgery, lifelong surveillance is mandatory. International guidelines recommend periodic biochemical assessment including: complete blood cell count, ferritin and iron studies, vitamin B12 and folate, calcium, vitamin D, and parathyroid hormone, fat-soluble vitamins, albumin and prealbumin, zinc, copper, selenium, and magnesium levels [5,19,20,53,57]. Routine supplementation typically includes high-potency multivitamins, calcium citrate with vitamin D, iron, vitamin B12, and additional fat-soluble vitamins when indicated. Adherence to supplementation is a major determinant of long-term nutritional outcomes [38]. Patients undergoing BPD-DS often require aggressive supplementation strategies and close multidisciplinary follow-up. This procedure is now performed infrequently. Recent systematic reviews emphasize the importance of correcting preoperative micronutrient deficiencies before surgery, since many obese patients already present with subclinical nutritional deficits prior to bariatric intervention [58]. These aspects are discussed in greater detail in Section 6, Prevention and Management Strategies.

In summary, bariatric procedures are among the most effective interventions for severe obesity and metabolic disease; however, their benefits are accompanied by substantial nutritional consequences. The degree of malabsorption varies according to surgical anatomy (Table 2), with BPD-DS, currently performed infrequently, carrying the highest risk for severe macro- and micronutrient deficiencies. Long-term success depends not only on weight reduction but also on rigorous nutritional surveillance, patient adherence to supplementation, and multidisciplinary postoperative care. Advances in understanding gastrointestinal adaptation and microbiota changes may improve future strategies for minimizing nutritional complications while preserving metabolic efficacy [59].

## 5. Sarcopenia: Definition and Pathophysiology

### 5.1. Definition of Sarcopenia

The term “sarcopenia” was first introduced by Irwin Rosenberg in 1989 to describe the progressive decline in skeletal muscle mass, strength, and physical performance [60]. Functionally, patients with postoperative sarcopenia may experience reduced muscle strength, impaired mobility, increased fall risk, and diminished exercise tolerance. These effects are particularly concerning in older adults and postmenopausal women, populations already predisposed to age-related muscle decline [61].

Despite its widespread use, a universally accepted definition of sarcopenia has not yet been established. Over the years, several international expert working groups have proposed differing diagnostic definitions and criteria for the condition using diverse combinations of muscle strength, muscle quantity/quality, and physical performance [22]. The absence of a standardized definition makes it difficult to compare findings across studies involving different populations and diagnostic criteria, thereby contributing to the extensive variability observed in the reported prevalence of this condition.

The widely adopted European Working Group on Sarcopenia in Older People 2 (EWGSOP2) consensus defines sarcopenia primarily as a condition characterized by reduced muscle strength; low muscle strength indicates probable sarcopenia, reduced muscle quantity or quality confirms the diagnosis, and impaired physical performance identifies severe sarcopenia [12]. This hierarchical approach reflects the prognostic importance of muscle strength and avoids equating reduced muscle mass alone with sarcopenia.

In contrast, the European Society for Clinical Nutrition and Metabolism (ESPEN) and the European Association for the Study of Obesity (EASO) consensus, specifically for sarcopenic obesity (SO), combines excess adiposity with impaired muscle mass/function. Its diagnostic pathway includes assessment of muscle strength (e.g., handgrip or chair-stand tests) followed by body-composition evaluation, preferably with dual-energy X-ray absorptiometry (DEXA) or alternatively bioelectrical impedance analysis (BIA); computed tomography (CT) may be considered when clinically indicated. It also proposes staging according to the presence of obesity-related complications and muscle dysfunction [62]. The Sarcopenic Obesity Global Leadership Initiative (SOGLI) builds on ESPEN/EASO consensus to standardize SO screening, diagnosis and staging [62]. This will be discussed in further detail in Section 5.3.

Thus, EWGSOP2 is primarily a sarcopenia diagnostic framework, whereas ESPEN/EASO and SOGLI specifically address SO by incorporating excess adiposity. This distinction is particularly relevant after metabolic and bariatric surgery, where expected postoperative lean-mass loss should not automatically be classified as sarcopenia or SO. A 2026 review found that 72 studies had applied the SOGLI algorithm, but substantial heterogeneity in screening, body-composition assessment, and staging highlights the need for further standardization [63].

Variations in the methods used to assess muscle mass and function may contribute to heterogeneity across results. DEXA is widely used to assess body composition and provides reproducible estimates of appendicular lean mass with low radiation exposure, making it useful after bariatric surgery. However, DEXA lean mass is not equivalent to skeletal muscle mass, as it includes non-muscle tissue and body water, which may affect interpretation. Its prognostic value also remains controversial, with the Sarcopenia Definition and Outcomes Consortium (SDOC) finding inconsistent associations between DEXA-derived lean mass and adverse outcomes and therefore not recommending it as a defining component of sarcopenia [64]. BIA is an inexpensive, rapid, non-invasive, and accessible method suitable for repeated postoperative assessment. However, it indirectly estimates muscle and fat mass using device- and population-specific equations, making results sensitive to device differences, hydration, edema, and food intake. These limitations are particularly relevant after bariatric surgery due to rapid changes in hydration and nutritional status. Given the potential for greater BIA measurement error in individuals with obesity, future studies should consider the use and validation of obesity-specific or population-specific prediction equations to improve the accuracy of fat mass (FM) and appendicular lean mass (ALM) estimates [65,66]. This approach may reduce measurement bias and improve the clinical applicability of BIA in this population. CT provides high-resolution assessment of skeletal muscle quantity and quality/myosteatosis, making it valuable in obesity, in whom excess intramuscular adipose tissue may contribute to impaired muscle function despite relatively preserved muscle quantity. It also allows opportunistic assessment during clinically indicated imaging. However, routine use is limited by radiation exposure, cost, availability, and methodological heterogeneity, including differences in anatomical landmarks, segmentation, and cut-offs [67].

Regarding functional assessment, handgrip strength (HGS) is a simple, inexpensive, reproducible measure of muscle strength and is strongly associated with clinically relevant outcomes. EWGSOP2 therefore places low muscle strength at the beginning of its diagnostic algorithm. HGS is particularly useful in bariatric populations because it can be performed repeatedly without specialized imaging equipment. However, results are influenced by age, sex, body size, hand dominance, comorbidities, and testing protocol, and appropriate population-specific cut-offs are required [12]. Gait speed is a practical measure of physical performance and has strong associations with mobility limitation, falls, disability, hospitalization, and mortality. The SDOC specifically identified low gait speed and low grip strength as clinically meaningful components of sarcopenia [64]. However, gait speed is not a direct measure of muscle function and may be affected by osteoarthritis, neurological disease, cardiopulmonary limitation, balance disorders, and postoperative functional limitations. This is particularly relevant in patients with severe obesity, in whom musculoskeletal comorbidities may confound interpretation. Accordingly, ESPEN/EASO did not recommend gait speed as a mandatory component of the primary diagnostic pathway for sarcopenic obesity [16].

In summary, for patients undergoing MBS, no single measurement should be considered sufficient to diagnose sarcopenia. Lean-mass reduction measured by DXA or BIA should be interpreted in conjunction with muscle strength and physical performance. A practical approach is therefore to use HGS and/or chair-stand testing for functional assessment, combined with DEXA when body-composition quantification is clinically indicated; BIA may provide a more accessible alternative, while CT can be used opportunistically when clinically available. This multimodal approach is particularly important because postoperative reductions in lean mass may reflect physiological adaptation to weight loss rather than clinically relevant sarcopenia.

### 5.2. Mechanisms of Sarcopenia After Bariatric Surgery

Sarcopenia following MBS is increasingly recognized as a significant postoperative complication characterized by the progressive loss of skeletal muscle mass, strength, and physical performance. Although MBS procedures effectively induce weight reduction and improve metabolic comorbidities, the rapid and profound changes in body composition may predispose patients to accelerated muscle catabolism. Current evidence suggests that postoperative sarcopenia is a multifactorial condition involving nutritional deficiencies, metabolic adaptations, hormonal alterations, inflammation, reduced mechanical loading, and physical inactivity. A recent systematic review reported that muscle loss after MBS is largely proportional to weight loss and may not impair function [68]. Standardized measures and obesity-specific reference values are needed to distinguish adaptive from clinically significant muscle loss after MBS

#### 5.2.1. Rapid Weight Loss, Lean Mass and Muscle Function Reduction

One of the principal mechanisms underlying sarcopenia after bariatric surgery is the rapid reduction in total body weight during the early postoperative period. Although adipose tissue constitutes the primary target of weight loss, a substantial proportion of the reduction involves fat-free mass and skeletal muscle tissue. Studies indicate that the first 6–12 months after surgery are associated with the greatest decline in lean body mass due to severe caloric restriction, negative energy balance, and reduced protein intake [69].

The marked catabolic state observed after surgery promotes proteolysis and mobilization of amino acids from skeletal muscle in order to maintain essential metabolic processes. This effect may be exacerbated by postoperative nausea, vomiting, food intolerance, and poor dietary adherence, all of which reduce protein consumption. Inadequate protein intake impairs muscle protein synthesis and accelerates muscle degradation, particularly in older adults and patients with pre-existing obesity-related sarcopenia.

It may be useful to emphasize that postoperative loss of lean body mass should not automatically be equated with clinically relevant sarcopenia. A transient decline in lean mass is a well-recognized physiological response to major surgery, reflecting the acute inflammatory and catabolic response, reduced nutritional intake, immobilization, and increased protein turnover. Its extent may vary according to the magnitude and rate of weight loss, baseline body composition, age, sex, nutritional intake, and physical activity. These changes may be partially or completely reversible during recovery and, when considered in isolation, do not necessarily indicate sarcopenia.

In contrast, sarcopenia is a clinical condition primarily characterized by reduced muscle strength, with low muscle quantity and/or quality providing additional diagnostic evidence according to the current EWGSOP2 consensus [12]. Similarly, the Asian Working Group for Sarcopenia recommends integrating muscle strength, physical performance, and muscle mass rather than relying on muscle mass alone [70]. Accordingly, postoperative reductions in lean mass measured by DEXA or BIA should not necessarily be interpreted as evidence of functional deterioration. Instead, changes in lean mass should be considered in the context of overall alterations in body weight and composition and evaluated alongside functional measures, such as handgrip strength and gait speed, as well as muscle quality, nutritional status, and recovery trajectory.

This distinction is particularly relevant because postoperative alterations in hydration, inflammation, and extracellular fluid distribution may affect body-composition measurements and result in an apparent reduction in lean mass without necessarily reflecting a corresponding loss of contractile muscle tissue [19]. Moreover, the relationship between postoperative lean-mass loss and adverse clinical outcomes remains incompletely established. Therefore, longitudinal assessment integrating muscle quantity with muscle strength and physical performance may provide a more clinically meaningful interpretation of postoperative body-composition changes and help avoid conflating an expected physiological response to surgery with pathological sarcopenia.

#### 5.2.2. Protein Malnutrition and Amino Acid Deficiency

Protein deficiency represents one of the most important nutritional contributors to postoperative sarcopenia. Following bariatric surgery, especially malabsorptive procedures, patients frequently fail to achieve the recommended daily protein intake necessary to preserve muscle mass. Reduced gastric capacity, altered digestion, and intestinal bypass decrease both protein ingestion and amino acid absorption. Essential amino acids, particularly leucine, play a critical role in activating the mammalian target of rapamycin (mTOR) pathway, which regulates muscle protein synthesis. Impairment of this anabolic signaling pathway contributes to progressive muscle wasting [71]. Furthermore, insufficient dietary protein may reduce circulating insulin-like growth factor-1 (IGF-1), a key anabolic mediator involved in muscle maintenance and regeneration. The decline in IGF-1 signaling has been associated with reduced satellite cell activation and impaired muscle repair mechanisms.

#### 5.2.3. Hormonal and Metabolic Alterations

Bariatric surgery induces profound endocrine and metabolic changes that may influence skeletal muscle homeostasis. Alterations in gut hormones such as GLP-1, peptide YY, ghrelin, and leptin contribute to appetite suppression and weight loss but may also affect muscle metabolism indirectly. Reduced ghrelin levels, particularly after SG, may diminish anabolic signaling because ghrelin has recognized stimulatory effects on growth hormone secretion and muscle preservation [72].

Additionally, postoperative improvements in insulin sensitivity, while metabolically beneficial, are accompanied by reduced mechanical loading on skeletal muscle due to substantial body mass reduction. In individuals with severe obesity, skeletal muscle is chronically adapted to supporting elevated body weight; rapid unloading after surgery may therefore contribute to muscle atrophy through decreased muscle fiber stimulation and lower anabolic demand [73].

#### 5.2.4. Inflammation and Oxidative Stress

Chronic low-grade inflammation is a hallmark of obesity and contributes to impaired muscle metabolism [74]. MBS generally reduces systemic inflammation; however, transient postoperative inflammatory responses and persistent metabolic stress may continue to influence skeletal muscle catabolism. Elevated concentrations of inflammatory cytokines such as tumor necrosis factor-alpha (TNF-α) and interleukin-6 (IL-6) can activate proteolytic pathways, including the ubiquitin–proteasome system, leading to enhanced muscle protein breakdown [75].

Oxidative stress also plays an important role in postoperative sarcopenia. Mitochondrial dysfunction within skeletal muscle impairs energy production, reduces muscle efficiency, and promotes apoptosis of muscle fibers. Emerging evidence indicates that MBS may alter mitochondrial metabolism differently in adipose tissue and skeletal muscle, potentially contributing to muscular adaptation during rapid weight loss [76,77].

#### 5.2.5. Physical Inactivity and Reduced Mechanical Stimulus

Physical inactivity after bariatric surgery further accelerates muscle loss. Many patients experience reduced exercise tolerance, fatigue, or prolonged sedentary behavior during postoperative recovery. The absence of adequate resistance or strength training decreases mechanical stimulation of skeletal muscle, impairing hypertrophy signaling pathways and promoting disuse atrophy. Conversely, recent studies demonstrate that supervised resistance and combined exercise programs significantly attenuate postoperative muscle loss and improve muscle strength and functional performance. Exercise interventions appear to stimulate muscle protein synthesis, improve mitochondrial function, and enhance neuromuscular adaptation, thereby reducing the risk of sarcopenia after surgery [73].

#### 5.2.6. Micronutrient Deficiencies and Muscle Function

Micronutrient deficiencies frequently observed after MBS may also contribute to sarcopenia. Deficiencies in vitamin D, iron, vitamin B12, magnesium, selenium, and zinc negatively affect muscle metabolism, neuromuscular transmission, mitochondrial function, and muscle contraction. Vitamin D deficiency, in particular, has been associated with impaired muscle strength and increased risk of falls [11,78].

Iron deficiency may impair oxygen transport and muscular oxidative capacity, whereas deficiencies of selenium and magnesium may compromise mitochondrial activity and muscle energetics. These nutritional abnormalities are particularly common after procedures with a substantial malabsorptive component and may persist long-term without adequate supplementation [52].

#### 5.2.7. Gut–Muscle Axis and Microbiota Changes

Emerging research highlights the potential role of the gut–muscle axis in postoperative sarcopenia. Bariatric surgery profoundly alters gut microbiota composition, bile acid metabolism, and intestinal permeability. Changes in microbial diversity may influence skeletal muscle through modulation of inflammation, insulin sensitivity, amino acid metabolism, and mitochondrial function [79].

Certain microbial metabolites, including short-chain fatty acids, appear to exert anabolic and anti-inflammatory effects on skeletal muscle [80]. Dysbiosis following surgery may therefore contribute to altered muscle metabolism and impaired muscle preservation. Although this field remains under active investigation, the gut–muscle axis is increasingly considered an important mechanistic component of sarcopenia development after bariatric procedures [79].

In summary, the mechanisms of sarcopenia after bariatric surgery are complex and multifactorial. Rapid weight loss, inadequate protein intake, hormonal changes, inflammation, oxidative stress, micronutrient deficiencies, reduced physical activity, and alterations in gut microbiota collectively contribute to skeletal muscle loss and functional decline. While MBS remains highly effective for the treatment of severe obesity, preservation of muscle mass has become a critical component of long-term postoperative management. Early nutritional intervention, adequate protein supplementation, resistance exercise, and lifelong metabolic monitoring are essential strategies to minimize sarcopenia risk and optimize clinical outcomes after MBS [81].

### 5.3. Body Composition Changes and Sarcopenic Obesity

MBS induces substantial body-composition changes, characterized predominantly by fat-mass loss accompanied by a smaller, but clinically relevant, reduction in lean or fat-free mass. The extent of these changes varies according to the surgical procedure, magnitude and rate of weight loss, baseline body composition, and individual nutritional and lifestyle factors. A systematic review and meta-analysis of 59 studies reported that, at 12 months, MBS was associated with losses of approximately 8.1 kg in lean body mass, 8.2 kg in fat-free mass, and 3.2 kg in skeletal muscle mass, with more than half of lean-mass loss occurring within the first 3 months [44]. The predominant loss of adipose tissue nevertheless represents the major component of postoperative weight reduction. In a meta-analysis of 34 studies, RYGB was associated with a reduction of approximately 29 kg in fat mass and 10 kg in fat-free mass over the first year, although comparisons between surgical procedures suggest that differences in lean-mass loss are less pronounced than those observed for total weight and fat-mass reduction [82]. Similarly, randomized controlled trials have shown that RYGB produces greater weight and fat-mass loss than gastric banding, while changes in lean mass are broadly comparable [83].

Importantly, as already mentioned, postoperative lean-mass loss should not be interpreted in isolation as pathological muscle wasting or sarcopenia. Despite reductions in muscle mass, MBS may improve the muscle-to-fat ratio, myosteatosis, inflammation, and metabolic function [7]. Therefore, body-composition changes should be interpreted alongside muscle strength, physical performance, nutritional status, and functional capacity. This is particularly relevant when using DEXA or BIA, as measured lean mass is not synonymous with skeletal muscle mass and may be influenced by hydration and other non-muscular tissues [44]. Early nutritional and exercise interventions may help preserve muscle mass during the period of most rapid weight loss [29,44].

It is important to recognize that the rapidly expanding use of novel pharmacological therapies for diabetes and obesity, including semaglutide and tirzepatide, is associated with substantial changes in body composition. The new therapies do not inherently cause anatomical malabsorption, unlike bypass procedures, but reduced appetite, nausea/vomiting, and reduced food intake, which can potentially compromise protein and micronutrient intake. Recent evidence indicates that both pharmacological and surgical approaches to obesity treatment are associated with substantial fat-mass reduction accompanied by some degree of lean-mass loss. A systematic review of semaglutide studies demonstrated predominantly fat-mass loss, although reductions in lean mass were observed in some trials [84]. Similarly, a DEXA substudy of SURMOUNT-1 showed that approximately 75% of weight lost with tirzepatide was attributable to fat mass and approximately 25% to lean mass [85]. A network meta-analysis found that semaglutide and tirzepatide produced among the greatest reductions in body weight and fat mass but were also associated with significant reductions in lean mass. Interestingly, relative lean mass as a proportion of body weight was not necessarily adversely affected [86]. Importantly, a recent real-world single-center comparative study evaluating changes in body composition associated with bariatric surgery and newer GLP-1 receptor agonists found that both bariatric surgery and semaglutide/tirzepatide treatment were associated with significant reductions in fat mass, modest decreases in fat-free mass, and improvements in the fat-free mass-to-fat mass ratio over 24 months. However, these effects were more pronounced following bariatric surgery. Overall, both approaches effectively reduced adiposity over two years, with bariatric surgery showing a more favorable body composition profile due to greater fat mass loss [87]. These findings highlight that lean-mass reduction is not unique to bariatric surgery and should not automatically be interpreted as sarcopenia.

The increasing use of GLP-1-based therapies also creates opportunities for complementary or sequential treatment strategies. Recent clinical data suggest that GLP-1 receptor agonist therapy before MBS may facilitate preoperative weight and fat-mass reduction without additional lean-mass loss, although prospective studies with longer follow-up are required [88].

Sarcopenic obesity (SO) is a complex clinical condition characterized by the coexistence of excess adiposity and reduced skeletal muscle mass and/or function. In recent years, SO has emerged as a relevant complication both before and after bariatric surgery. Although MBS remains the most effective treatment for severe obesity and obesity-related metabolic disease, postoperative changes in body composition may predispose susceptible individuals to sarcopenia, SO, and functional impairment. Recent evidence suggests that the interaction between obesity, chronic inflammation, anabolic resistance, and postoperative nutritional deficiencies contributes significantly to the development of SO after MBS [89].

**Figure 1:**
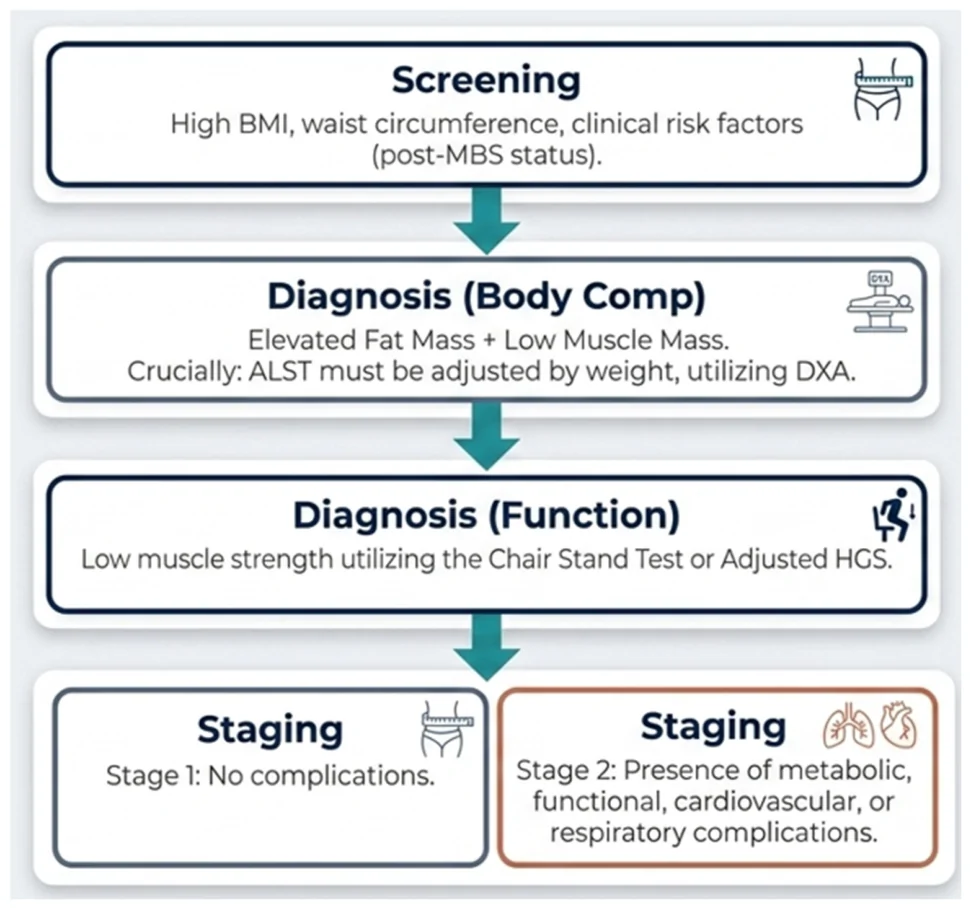
The SOGLI (Sarcopenic Obesity Global Leadership Initiative) criteria. A consensus-based definition and diagnostic framework for sarcopenic obesity developed by ESPEN and EASO. Launched in 2022/2023, it aims to standardize the diagnosis by distinguishing sarcopenic obesity within populations at high risk [62]. EASO: European Association for the Study of Obesity; ESPEN: The European Society for Clinical Nutrition and Metabolism. Illustration created using Gemini Notebook.

According to the 2022 ESPEN/EASO consensus, SO is currently defined as the simultaneous presence of obesity and impaired skeletal muscle function associated with low muscle mass. Diagnostic evaluation typically includes assessment of muscle strength (e.g., handgrip strength), body composition by DEXA or BIA, and physical performance tests [61]. The SOGLI criteria [62] are a consensus-based framework developed by the ESPEN and EASO to standardize the diagnosis of SO through the combined assessment of body composition and muscle function, which are shown in Figure 1.

In patients undergoing MBS, the diagnosis of SO is particularly challenging because rapid postoperative weight loss can mask progressive muscle depletion. Several studies have shown wide variability in SO prevalence depending on the diagnostic criteria used. A 2024 study in candidates for bariatric surgery demonstrated that prevalence estimates vary substantially according to the selected cut-off values and body composition method employed [90].

The mechanisms underlying SO after bariatric surgery are multifactorial and involve metabolic, hormonal, inflammatory, and nutritional pathways [15]. Obesity itself promotes skeletal muscle dysfunction through chronic low-grade inflammation, insulin resistance, mitochondrial dysfunction, and adipose tissue infiltration within muscle fibers. These abnormalities may persist even after successful surgical weight reduction [7]. Bariatric procedures such as RYGB and SG induce substantial reductions in fat and lean mass loss, especially during the first postoperative year. Muscle loss may account for approximately 20–30% of total weight reduction after surgery [15].

The rapid catabolic state following surgery is driven by several factors including: (i) markedly reduced caloric and protein intake; (ii) decreased mechanical loading due to weight loss; (iii) hormonal changes affecting muscle protein synthesis; (iv) micronutrient deficiencies (vitamin D, iron, vitamin B12, thiamine); and (v) inadequate physical activity during recovery [7,15]. Protein malabsorption and anabolic resistance are particularly important contributors. Reduced amino acid availability impairs activation of the mTOR pathway, which is essential for muscle protein synthesis. In addition, postoperative inflammation and altered gut-derived hormonal signaling may exacerbate muscle catabolism [91]. It could be speculated that patients with SO prior to bariatric surgery may experience different postoperative outcomes compared with obese patients without sarcopenia. However, a recent systematic review and meta-analysis evaluated the efficacy and safety of metabolic and bariatric surgery in patients with SO. Seven studies including 1704 patients were analyzed. Compared with non-SO patients, the SO group showed greater reductions in body weight and BMI, with similar trends in total and excess weight loss. Remission of comorbidities and postoperative complication rates were comparable between groups. Overall, MBS appeared safe and effective also in patients with SO, providing moderate weight loss and metabolic improvement without increased postoperative risk [92].

SO after bariatric surgery is associated with several adverse outcomes, including reduced physical performance, frailty, decreased quality of life, impaired metabolic adaptation, and increased risk of weight regain [61]. It has been shown that fracture risk is increased in patients who undergo bariatric surgery compared with non-operated individuals [93]. Bone health after bariatric surgery is also influenced by additional factors, including alterations in endocrine signals from the pancreas, adipose tissue, and gastrointestinal tract—such as insulin, leptin, estradiol, and ghrelin, among others [94]. Emerging evidence also suggests a relationship between SO and metabolic dysfunction-associated steatotic liver disease. A 2024 cohort study involving 972 bariatric surgery patients demonstrated that SO was associated with more severe hepatic and metabolic phenotypes before surgery, including insulin resistance and steatohepatitis. However, bariatric surgery still produced significant metabolic improvements in these patients, indicating that surgery remains beneficial despite the presence of SO [89].

The evolution of body composition after bariatric surgery is dynamic. During the first 6–12 months, rapid fat loss is accompanied by substantial reductions in fat-free mass. Nevertheless, some investigators argue that bariatric surgery may ultimately improve the muscle-to-fat ratio and reduce the overall burden of SO despite lean mass reductions. In fact, recent narrative reviews indicate that skeletal muscle insulin sensitivity and mitochondrial function often improve after surgery, potentially counterbalancing some deleterious effects of muscle mass loss suggesting that postoperative changes should be interpreted not only quantitatively but also functionally [7].

**Figure 2:**
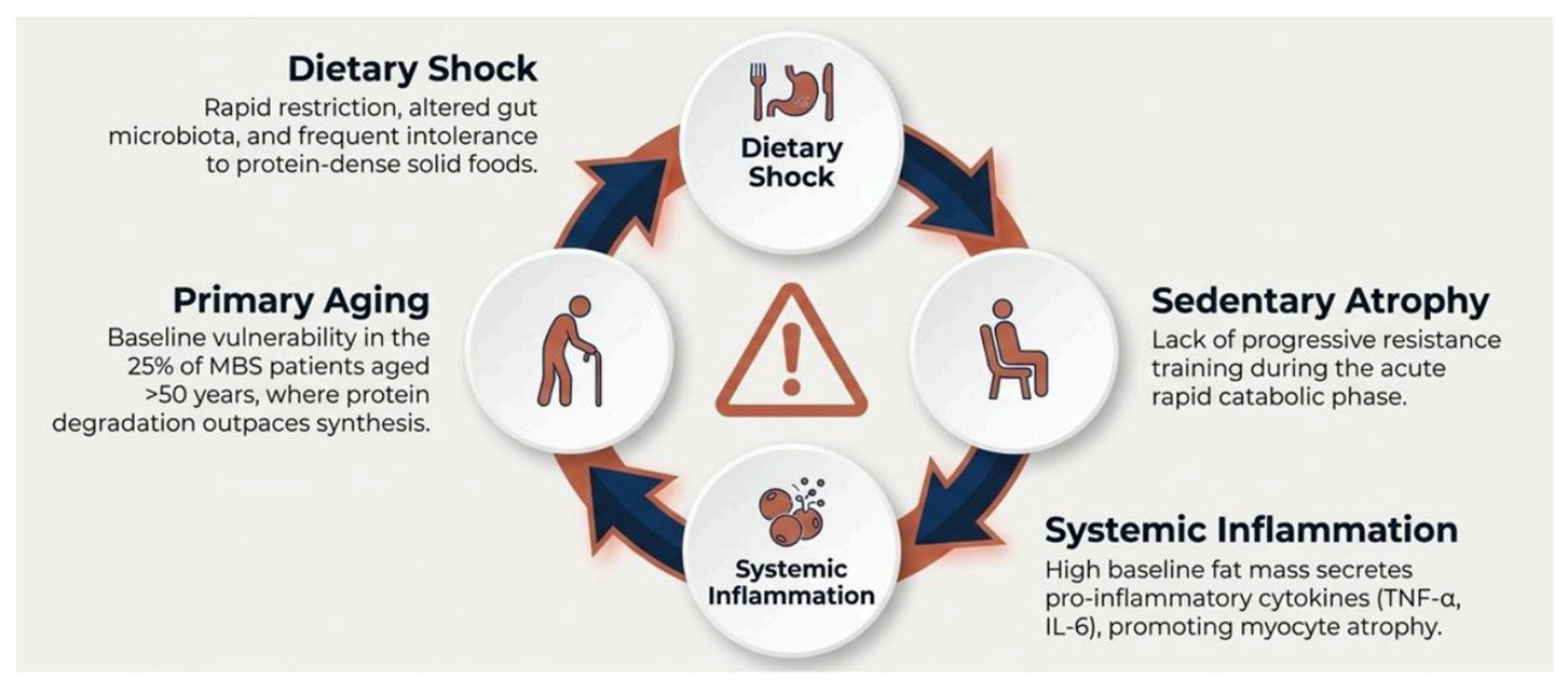
The vicious circle of sarcopenic obesity. Insulin resistance and chronic inflammation create a self-fulfilling loop that propagates muscle loss while preserving adipose tissue. MBS: metabolic bariatric surgery; TNF: tumor necrosis factor alpha; IL-6: interleukin 6. Illustration created using Gemini Notebook.

Figure 2 illustrates the vicious cycle of sarcopenic obesity, in which insulin resistance and chronic low-grade inflammation form a reinforcing loop that promotes skeletal muscle loss while maintaining adipose tissue.

### 5.4. Bone Health and Osteosarcopenia

Bone health should be considered an integral component of body-composition assessment after MBS, particularly because concomitant losses of muscle mass and BMD may contribute to an osteosarcopenic phenotype. MBS is associated with increased bone turnover and reductions in BMD, driven by rapid weight loss, reduced mechanical loading, alterations in calcium and other nutrients essential for bone health, impaired vitamin D metabolism, and endocrine adaptations [95,96,97]. A 2024 systematic review and meta-analysis confirmed significant differences in short-term BMD and calciotropic hormones between RYGB and SG, with greater reductions in BMD, lower vitamin D and higher PTH levels after RYGB [95]. Similarly, recent evidence indicates that skeletal deterioration may involve not only areal BMD but also alterations in bone microarchitecture, supporting a more comprehensive assessment of skeletal health after MBS [98]. These effects are particularly relevant after procedures with a substantial malabsorptive component, which carry a greater risk of micronutrient malabsorption and metabolic bone disease [96,99]. The European Calcified Tissue Society has reported a 21–44% increase in overall fracture risk following bariatric surgery, with the highest risk observed after procedures with a malabsorptive component, including RYGB and BPD [96]. It should be noted that BPD is currently an uncommon bariatric procedure. Treatment of osteoporosis after MBS requires correction of calcium and vitamin D deficiencies before therapy. When treatment is indicated, intravenous bisphosphonates, particularly zoledronic acid, are generally preferred over oral agents because of concerns regarding gastrointestinal absorption after bariatric surgery [53,96]. Denosumab may be considered when bisphosphonates are contraindicated or poorly tolerated, but careful monitoring of calcium and vitamin D is essential due to the risk of hypocalcemia [96,100]. Evidence specific to post-bariatric patients remains limited; therefore, treatment should be individualized according to BMD, fracture history, and overall fracture risk [53,96].

Accordingly, longitudinal evaluation of bone health—including calcium, 25-hydroxyvitamin D, phosphorus, PTH, and, when clinically indicated, BMD by DEXA—should complement assessment of muscle mass, strength, and physical performance. However, the available evidence remains heterogeneous, and the clinical significance of simultaneous changes in bone and muscle compartments should be interpreted in the context of individual fracture risk and functional status.

## 6. Prevention and Management Strategies

### 6.1. Protein Supplementation

Current evidence supports adequate intake of high-quality protein after MBS to preserve lean body mass, promote wound healing, and minimize the risk of protein malnutrition during rapid weight loss. Protein intake should be tailored to the type of MBS. For SG, RYGB, and AGB, current recommendations generally suggest 60–80 g/day or approximately 1.0–1.5 g/kg ideal body weight [100,101]. In contrast, patients undergoing procedures with greater hypoabsorptive potential, such as BPD/DS, OAGB, or SADI-S, may require ≥90 g/day and, in selected cases, up to 2.1 g/kg ideal body weight/day because of the greater risk of protein-energy malnutrition [101]. Recent reviews emphasize, however, that these targets are largely based on expert consensus and limited direct evidence; therefore, protein requirements should be individualized according to age, sex, body composition, weight-loss trajectory, nutritional status, and surgical procedure [102,103]. Whey-protein supplementation is commonly preferred because of its high leucine content and anabolic potential. Emerging 2025 evidence suggests protein supplementation may improve fat-free mass and muscle preservation after surgery, although the optimal dose and timing remain under investigation [104,105].

### 6.2. Micronutrient Supplementation

Updated guidelines emphasize that lifelong micronutrient supplementation and biochemical monitoring are essential after bariatric surgery because reduced intake and malabsorption increase the risk of deficiencies. Recommendations from the ASMBS support routine use of a bariatric-specific multivitamin containing thiamine (≥12 mg/day), vitamin B12 (350–500 μg/day monthly intramuscular supplementation), folate (400–800 μg/day), iron (18–60 mg/day depending on sex and procedure), vitamin D3 (~3000 IU/day titrated to serum levels), calcium citrate (1200–2400 mg/day depending on procedure), and additional fat-soluble vitamins, zinc, and copper when indicated. More malabsorptive procedures such as RYGB and BPD-DS require higher supplementation doses, parenteral supplementation and closer laboratory surveillance. The recent literature continues to highlight vitamin D, iron, zinc, and vitamin K deficiencies as among the most prevalent long-term complications, reinforcing the importance of individualized supplementation and lifelong follow-up [105].

### 6.3. Physical Exercise

Current evidence supports incorporating structured resistance exercise early after bariatric surgery to preserve lean body mass, improve muscle strength, and enhance functional recovery during rapid postoperative weight loss [73]. Recent systematic reviews recommend combining resistance training with aerobic exercise, ideally beginning once postoperative recovery is complete (typically 4–8 weeks after surgery), with sessions performed 2–3 times per week targeting major muscle groups using progressive overload principles. Moderate-intensity programs involving 2–3 sets of 8–12 repetitions have shown benefits for muscle strength, physical function, and mitigation of sarcopenia risk after procedures such as SG and RYGB [106]. This is in line with most studies and expert-based recommendations suggesting progressively achieving 150–300 min/week of supervised or semi-supervised physical activity moderate-intensity aerobic activity, with full-body resistance training initiated approximately 4–6 weeks after surgery and performed at least twice weekly to minimize sarcopenia and improve overall physical function and quality of life after surgery [106,107,108]. High-intensity and abdominal exercises are generally postponed until approximately 8–12 weeks postoperatively, depending on surgical recovery [108]. EASO recommendations similarly emphasize 150–200 min/week of moderate aerobic exercise and the integration of resistance training to preserve lean mass and improve physical fitness [109,110] (Table 3).

A recent systematic review and meta-analysis on trials in adults undergoing MBS assessed the effects of structured exercise interventions before or after surgery, including 33 studies among 1545 participants. The structured intervention significantly reduced body weight and BMI, particularly with aerobic exercise, while preserving fat-free mass, especially in combined exercise programs. Muscle strength improved most with resistance training, alongside gains in functional and aerobic capacity. Physical activity increased overall, although step count and resting metabolic rate showed no significant changes. Thus, early postoperative supervised combined exercise programs are recommended and should be maintained for at least six months when clinically appropriate [107].

In addition, the evidence supports the inclusion of structured postural and exercise rehabilitation programs after MBS to improve functional mobility, musculoskeletal alignment, balance, and preservation of lean body mass. Therefore, programs may combine low- to moderate-intensity aerobic exercise and resistance training with flexibility and postural stabilization exercises beginning early in the postoperative period and progressing gradually according to tolerance. Programs emphasizing core strengthening, spinal stabilization, gait retraining, stretching, and proprioceptive exercises appear particularly beneficial for correcting postural alterations associated with rapid weight loss and pre-existing obesity-related biomechanical dysfunctions [106,107]. Exercise prescription should nevertheless be individualized according to preoperative fitness, comorbidities, postoperative recovery, nutritional status, and functional capacity.

**Figure 3:**
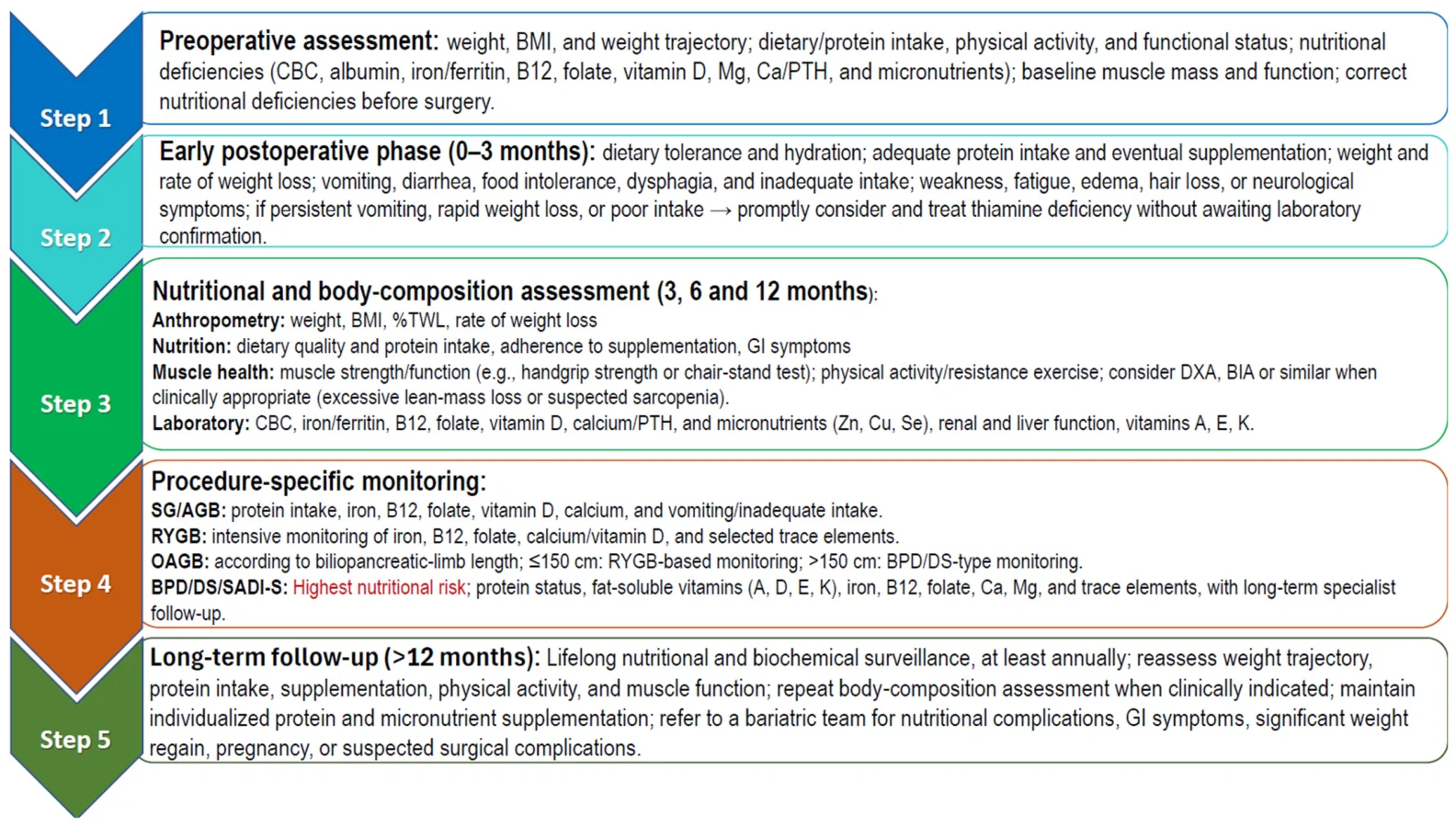
Proposed clinical practical algorithm for nutritional and muscle-health monitoring after metabolic and bariatric surgery. AGB: adjustable gastric band; BIA: bioelectrical impedance analysis; BMI: body mass index; BPD-DS: biliopancreatic diversion with duodenal switch Ca: calcium; CBC: complete blood count; Cu: copper; DEXA: dual-energy X-ray absorptiometry; GI: gastrointestinal; Mg: magnesium; OAGB: one anastomosis gastric bypass; PTH: parathyroid hormone; RYGB: Roux-en-Y gastric bypass; SADI-S: single anastomosis duodenal-ileal bypass with sleeve; Se: selenium; SG: sleeve gastrectomy; %TWL: percentage total weight loss; Zn: zinc.

Figure 3 proposes a clinical practical pathway that integrates nutritional assessment, body-composition surveillance, laboratory monitoring, and escalation according to the type of procedure and clinical risk. This is consistent with current bariatric nutritional guidance, which recommends specialist follow-up for at least the first 2 years and lifelong nutritional surveillance thereafter.

Risk stratification is essential for individualizing postoperative follow-up and identifying patients who require early intervention. Nutritional and muscle-related complications are not uniform across patients or procedures; therefore, monitoring intensity should be adapted to the patient’s surgical anatomy, nutritional status, rate of weight loss, protein intake, gastrointestinal symptoms, and muscle function. This approach allows low-risk patients to continue standard surveillance while enabling earlier nutritional, functional, and multidisciplinary intervention in intermediate- and high-risk patients, potentially preventing progression to severe protein-energy malnutrition, sarcopenia, or irreversible micronutrient complications. In particular, persistent vomiting, rapid weight loss, inadequate protein intake, edema, hypoalbuminemia, marked lean-mass loss, or neurological symptoms should be considered red flags requiring prompt evaluation and intensified management. Importantly, protein-energy malnutrition may develop even years after surgery, particularly after highly malabsorptive procedures, highlighting the need for lifelong risk-based surveillance. Table 4 summarizes the key criteria for postoperative risk stratification and identification of patients requiring different levels of clinical monitoring and intervention.

## 7. Future Directions and Research Gaps

Future research on the nutritional and sarcopenic consequences of bariatric surgery should prioritize standardized diagnostic criteria, long-term monitoring strategies, and individualized interventions to prevent excessive lean mass loss and micronutrient deficiencies after surgery. Although bariatric procedures effectively improve obesity-related comorbidities, recent evidence shows that postoperative sarcopenia and SO remain frequent complications, particularly following rapid weight loss and inadequate protein intake. Current research gaps include inconsistent definitions and assessment of sarcopenia, limited long-term (>5 years) follow-up studies, and insufficient evidence regarding optimal protein supplementation, resistance exercise protocols, and micronutrient management according to surgical technique. Emerging fields of investigation include gut microbiota modulation, inflammatory and metabolic biomarkers, muscle quality imaging, and AI-based body composition analysis for early risk prediction and personalized postoperative care. Recent systematic reviews also emphasize the importance of multidisciplinary follow-up integrating nutrition, physical rehabilitation, and functional assessment to improve long-term musculoskeletal outcomes after bariatric surgery.

**Figure 4:**
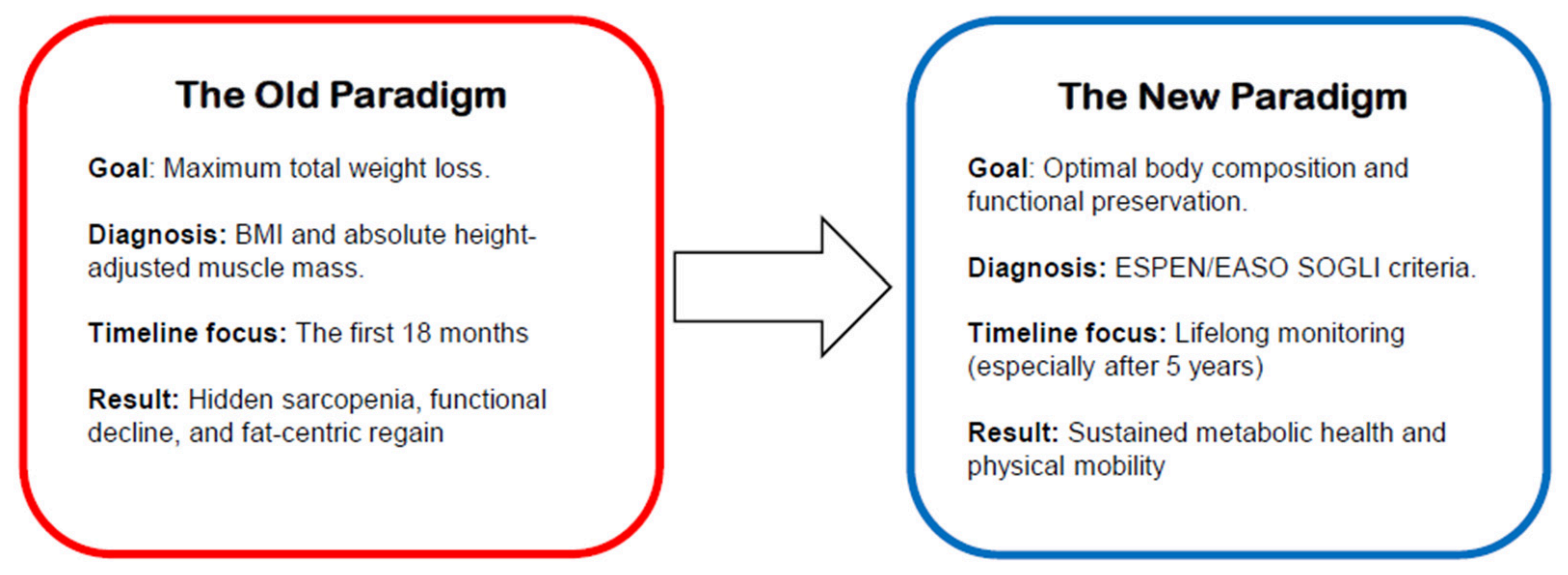
The paradigm shift in bariatric surgery care. BMI: body mass index; EASO: European Association for the Study of Obesity; ESPEN: The European Society for Clinical Nutrition and Metabolism; SOGLI: Sarcopenic Obesity Global Leadership Initiative.

The paradigm shift in bariatric surgery care reflects the transition from a procedure-centered approach focused primarily on weight loss toward a comprehensive, multidisciplinary model aimed at long-term metabolic, nutritional, functional, and quality-of-life outcomes (Figure 4). Contemporary MBS management increasingly recognizes obesity as a chronic, multifactorial disease requiring continuous follow-up that integrates surgical care with nutritional monitoring, physical rehabilitation, psychological support, and assessment of body composition and muscle health. This evolving paradigm also emphasizes the prevention and early detection of complications such as sarcopenia, SO, micronutrient deficiencies, and functional decline, particularly during the rapid postoperative weight-loss phase. Advances in precision medicine, personalized nutrition, exercise prescription, digital health monitoring, and AI-assisted body composition analysis are further reshaping postoperative care, promoting individualized treatment strategies that extend beyond weight reduction to optimize metabolic health, physical function, and long-term patient outcomes.

## 8. Conclusions

In conclusion, bariatric surgery induces profound and sustained metabolic benefits but is also associated with clinically relevant nutritional deficiencies and loss of lean body mass, which may contribute to sarcopenia and, in some cases, sarcopenic obesity. These effects are driven by a combination of reduced energy and protein intake, altered gastrointestinal anatomy, hormonal changes, and increased inflammatory and catabolic stress during rapid weight loss. The clinical consequences extend beyond body composition, potentially affecting physical function, quality of life, and long-term health outcomes if not adequately addressed. Effective management requires a lifelong, multidisciplinary approach integrating individualized nutritional supplementation, structured resistance and aerobic exercise, and regular assessment of body composition and muscle function. Future strategies should focus on standardized diagnostic criteria, early risk stratification, and precision-based interventions to preserve skeletal muscle while sustaining metabolic improvements after surgery.

## Figures and Tables

**Table 1:** Parametric classification of metabolic and bariatric surgery based on anatomical targets and surgical extent.

Non-Resectional Bariatric Surgery					Resectional Bariatric Surgery			
Isolate Gastric BS		Gastrointestinal Bypass BS		Isolated Intestinal Bypass BS	Isolated Gastric BS	Gastro-Intestinal Bypass BS		
VBG	AGB	RYGB	OAGB	Jejuno-Ileostomy	Sleeve gastrectomy	SASI	SADI-S	Resectional bypass surgery

Abbreviations: AGB: adjustable gastric band; BS: bariatric surgery; OAGB: one anastomosis gastric bypass; RYGB: Roux-en-Y gastric bypass; SADI-S: single anastomosis duodenal-ileal bypass with sleeve; SASI: sleeve with loop bipartition; VBG: vertical banded gastroplasty.

**Table 2:** Comparative nutritional consequences and recommendations in diverse bariatric surgical procedures [40,44,45,46,47,48].

Bariatric Procedure	Expected Weight Loss	Lean Mass Reduction	Protein Deficiency Risk	Main Micronutrient Deficiencies	Recommended Laboratory Monitoring	Long-Term Nutritional Management
Sleeve gastrectomy (SG)	Moderate–high: ~25–30% TBWL; typically, ~50–70% EWL	Moderate; lean mass may account for ~20–30% of total weight lost, with variability according to age, sex, protein intake and physical activity	Low–moderate, but clinically relevant, particularly with inadequate protein intake, vomiting, or poor dietary tolerance	Iron, vitamin B12, folate, vitamin D, calcium; thiamine with vomiting; zinc/copper less commonly	CBC, ferritin/iron studies, B12, folate, 25-OH vitamin D, calcium/PTH, renal and liver function; consider zinc/copper and thiamine according to symptoms/risk	Lifelong multivitamin/mineral supplementation; protein-rich diet, generally targeting ~60–80 g/day or individualized according to body size and clinical status; calcium/vitamin D and B12 supplementation; regular dietetic follow-up.
Roux-en-Y gastric bypass (RYGB)	High: ~30–35% TBWL; ~60–80% EWL	Moderate–high; lean tissue loss may be substantial during rapid weight loss	Moderate; greater than SG because of reduced intake plus bypass of proximal small intestine	Iron, vitamin B12, folate, calcium, vitamin D; zinc, copper and thiamine; vitamins A/E/K may occur, particularly with poor intake or complications	CBC, ferritin/iron panel, B12 ± MMA, folate, vitamin D, calcium/PTH, renal/liver function; periodic zinc/copper; selenium and fat-soluble vitamins when clinically indicated; thiamine with vomiting/rapid weight loss	Lifelong multivitamin/mineral supplementation; higher attention to iron, B12, calcium/vitamin D; adequate protein intake; individualized supplementation according to laboratory results; annual lifelong biochemical surveillance after the initial intensive follow-up period.
One-anastomosis gastric bypass (OAGB/MGB)	High: generally, ~30–35% TBWL; often comparable to or somewhat greater than RYGB	Moderate–high	Moderate–high, influenced by biliopancreatic limb length and degree of malabsorption	Iron, B12, folate, calcium, vitamin D; zinc, copper, selenium; fat-soluble vitamins with longer biliopancreatic limbs	CBC, ferritin/iron studies, B12, folate, vitamin D, calcium/PTH, renal/liver function; zinc, copper and selenium; vitamins A/E/K particularly when a longer biliopancreatic limb is used or symptoms occur	Lifelong comprehensive supplementation and annual biochemical monitoring; protein intake should be closely monitored; nutritional surveillance should be intensified when the biliopancreatic limb is long or when malabsorptive complications occur.
Single-anastomosis duodeno-ileal bypass with sleeve gastrectomy (SADI-S)	Very high: ~30–40% TBWL; generally, between RYGB and BPD/DS, depending on technique	High	High, although potentially somewhat lower than conventional BPD/DS depending on common-channel length	Iron, B12, folate, calcium, vitamin D, vitamins A/E/K, zinc, copper and selenium	Similar to BPD/DS: CBC, ferritin/iron, B12, folate, vitamin D, calcium/PTH, albumin/protein, vitamins A/E/K, zinc, copper, selenium, renal/liver function	Lifelong high-protein intake and comprehensive supplementation; close monitoring for protein malnutrition and fat-soluble vitamin/trace-element deficiencies. The more malabsorptive the configuration, the more intensive the surveillance required.
Biliopancreatic diversion with duodenal switch (BPD/DS)	Very high: ~35–45% TBWL; generally, the greatest weight loss among established procedures	High; substantial lean tissue loss can occur during rapid weight reduction	High–very high; greatest risk of protein-energy malnutrition among established bariatric procedures, although currently performed infrequently	Iron, B12, folate, calcium, vitamin D, vitamins A/E/K, zinc, copper, selenium; deficiencies may be multiple and severe	CBC, ferritin/iron panel, B12, folate, vitamin D, calcium/PTH, albumin/total protein; vitamins A, E and K; zinc, copper, selenium; renal/liver function; consider bone-density assessment and additional tests according to symptoms	Lifelong, intensive nutritional management; high-protein diet and substantially greater supplementation than restrictive procedures; lifelong multivitamin/mineral, calcium/vitamin D, iron and B12, with regular fat-soluble vitamin and trace-element monitoring. Long-term specialist follow-up is essential.
Adjustable gastric banding (AGB)	Low–moderate: ~15–25% TBWL; generally, less than SG or bypass	Low–moderate; primarily related to overall weight loss and inadequate protein intake	Low; increased with vomiting, food intolerance, or inadequate intake	Iron, vitamin B12, folate, vitamin D, calcium; deficiencies generally less frequent than after bypass	CBC, ferritin/iron studies, B12, folate, calcium, vitamin D/PTH, electrolytes; assess thiamine if prolonged vomiting or rapid weight loss	Lifelong multivitamin/mineral supplementation; adequate protein intake; dietary counseling to maintain food quality and avoid maladaptive eating; monitor for vomiting and intolerance. Annual nutritional assessment is recommended.

Abbreviations: CBC: complete blood count; EWL: excess weight loss; MMA: methylmalonic acid; PTH: parathyroid hormone; TBWL: total body weight loss.

**Table 3:** Exercise recommendations after metabolic bariatric surgery.

Organization/Evidence Source	Aerobic Exercise	Resistance Training	Timing/Progression	Main Objectives
Expert-based MBS recommendations, 2025 [108]	Progressively increase to 150–300 min/w of moderate-intensity cardiovascular activity	Full-body RT 2 non- consecutive days/w, progressively introduced	Walking/short-distance ambulation from the day of surgery; progressively increase daily activity during the first month; RT from 4 to 6 weeks; high-intensity and abdominal exercises from 8–12 weeks	Improve fitness and help preserve lean mass
EASO Physical Activity Working Group, 2021 [109]	Approximately 150–200 min/w of moderate- intensity aerobic exercise	Combine aerobic and resistance exercise; RT is particularly relevant for preserving lean mass	Gradual, individualized progression according to functional capacity	Preserve lean mass, improve cardiorespiratory and muscular fitness, and support weight management
EASO post-bariatric recommendations, 2018 [110]	≥150 min/w, progressing toward 300 min/w of moderate aerobic activity	2–3 sessions/w	Begin after recovery from surgery; walking is recommended as an accessible initial modality	Weight maintenance, physical fitness, and prevention of lean-mass loss

Abbreviations: EASO: European Association for the Study of Obesity; MBS: metabolic and bariatric surgery; RT: resistance training; w: week.

**Table 4:** Postoperative risk stratification.

Risk Category	Typical Characteristics	Management
Low risk	Adequate protein intake, stable/improving nutritional markers, preserved muscle function, no significant gastrointestinal symptoms	Continue standard supplementation, dietary counseling, physical activity/resistance exercise and scheduled follow-up
Intermediate risk	Excessive/rapid weight loss, inadequate protein intake, declining muscle mass or strength, borderline micronutrient abnormalities, persistent gastrointestinal symptoms	Intensify dietetic follow-up; optimize protein and supplementation; initiate resistance exercise; repeat biochemical/body-composition assessment sooner
High risk	Protein malnutrition, hypoalbuminemia, edema, marked lean-mass loss, significant micronutrient deficiencies, persistent vomiting/diarrhea, severe weakness or neurological symptoms	Urgent multidisciplinary assessment; correct deficiencies; investigate anatomical/functional causes; consider specialized nutritional support and referral to the bariatric center

## Data Availability

No new data were created or analyzed in this study. Data sharing is not applicable to this article.

## Abbreviations

The following abbreviations are used in this manuscript:

AGB	adjustable gastric band
BIA	bioelectrical impedance analysis
BMI	body mass index
BPD	biliopancreatic diversion
BPD-DS	biliopancreatic diversion with duodenal switch
BS	bariatric surgery
DEXA	dual-energy X-ray absorptiometry
EASO	European Association for the Study of Obesity
ESPEN	European Society for Clinical Nutrition and Metabolism
GLP-1	glucagon-like peptide-1
IL-6	interleukin-6
MBS	Metabolic and bariatric surgery
OAGB	one-anastomosis gastric bypass
RYGB	Roux-en-Y gastric bypass
SADI-S	single anastomosis duodenal-ileal bypass with sleeve
SASI	sleeve with loop bipartition
SG	sleeve gastrectomy
SO	sarcopenic obesity
SOGLI	Sarcopenic Obesity Global Leadership Initiative
TNF-α	tumor necrosis factor-alpha
VBG	vertical banded gastroplasty
